# The changing context of Health in All Policies in Finland since the 8th Global Conference on Health Promotion in Finland

**DOI:** 10.1093/heapro/daaf239

**Published:** 2026-01-13

**Authors:** Eeva Ollila, Leena Tervonen, Meri Koivusalo

**Affiliations:** Cancer Society of Finland, Mäkelänkatu 2, 00500 Helsinki, Finland; Faculty of Social Sciences, Tampere University, Arvo Ylpönkatu 34, 33520 Tampere, Finland; Cancer Society of Finland, Mäkelänkatu 2, 00500 Helsinki, Finland; Cancer Society of Finland, Mäkelänkatu 2, 00500 Helsinki, Finland; Faculty of Social Sciences, Tampere University, Arvo Ylpönkatu 34, 33520 Tampere, Finland

Comprehensive public health policies were developed and initiated in Finland in early 1970s (Talousneuvosto 1972). The first steps were taken to prevent road accidents, work-related diseases and accidents, and smoking-related diseases. Coronary heart diseases were tackled through improving diet, reduce smoking, and lowering high-blood pressure, in particular in the higher hit province of North Karelia, where the North Karelia project was started in 1972 to influence people’s lifestyles.

Intersectoral public health policy was further developed over the consecutive decades in close collaboration with the World Health Organization (WHO). Finland was a pioneer country in the Health for All programme in the WHO European Region and an active participant in the Ottawa Conference contributing to the drafting of the Charter, Healthy Public Policy in particular. Implementation of intersectoral health policies was based on government-approved national public health programmes (Melkas 2013).

In the 1990s, legislation on intersectoral responsibility and accountability for health was strengthened, including establishing the legal basis for an intersectoral advisory board for public health and intersectoral public health reports. In the 2000s, the local level was required by law to take health into account in all their policies, assess health impacts on various population groups, produce, and discuss well-being reports, as well as to use indicators to monitor development (Melkas 2013).

Joining the European Union (EU) in 1995 changed the national health policy landscape, as many of the major determinants of health had substantial EU level competence (Melkas 2013). The legal basis of health protection in the EU was first established in the Maastricht Treaty of 1992 (Article 129), strengthened for all policies in Amsterdam Treaty of 1997 (Article 152), and remains consolidated in the Lisbon Treaty 2007 (Article 168).

In 2006, the Finnish EU Presidency sought to strengthen the operationalization of the article in the EU and its member states by establishing Health in All Policies (HiAP) as its main health theme and producing a book on the concept (Ståhl *et al*. 2006). In 2013, the WHO and the Finnish Ministry of Social Affairs and Health organized the 8th Global Conference on Health Promotion (8GCHP) on HiAP in Helsinki. Linked to the conference a book exploring the experiences in and opportunities for HiAP implementation across the globe was produced (Leppo *et al*. 2013).

The conference statement of the 8GCHP defined HiAP as ‘an approach to public policies across sectors that systematically takes into account the health implications of decisions, seeks synergies, and avoids harmful health impacts in order to improve population health and health equity. It improves accountability of policymakers for health impacts at all levels of policy-making. It includes an emphasis on the consequences of public policies on health systems, determinants of health and well-being’. It also stated that while governments have a range of priorities in which health and equity do not automatically gain precedence over other policy objectives, governments should ensure that health considerations are transparently taken into account in policymaking and to open up opportunities for co-benefits across sectors and society at large (Health in All Policies 2014).

The preparation of the HiAP theme for the EU Presidency in 2006 as well as for the Global Health Promotion Conference in 2013 served as an inspiration for strengthening HiAP implementation within Finland. For example, annual intersectoral 2-day seminars to discuss intersectoral issues were established. Health and health equity aspects were further strengthened in the guidance for integrated impact assessments.

At the same time, health and health sector were increasingly seen through the lens of economic policies and potential to improve employment, extend working careers, enable economic growth, and enhance internal security (Sosiaali- ja terveysministeriö 2014). Special emphasis was paid on the potential of health services and technologies for economic growth (Ministry of Economic Affairs and Employment 2014).

Over the recent years a major reform of the health and social services has taken place, shifting the contemporary health policy focus on reforming the health care structures and services. Within this reform the public health law was replaced by new laws on social and health care and their organizing, and the intersectoral advisory board on public health lost its legislative basis and was abolished. The legal basis for the intersectoral public health reports had been dismantled already earlier.

While overall focus on HiAP has become weaker, issue-based policies and intersectoral bodies, including on nutrition and physical activity, have been more resilient or as regards that of physical activity even strengthened. Intersectoral work on mental health promotion has grown in recent years. Tobacco policies have been relatively successful as regards cigarettes, while the use of snus and more recently that of e-cigarettes and nicotine pouches have increased especially among young people (Ollila and Ruokolainen 2025). This is due to the fact that product development and marketing have been targeted towards young people, the growing importance of digital marketing as well as to the difficulties in putting proper regulations in place both nationally and in the context of the EU (Ollila 2020, Salokannel and Ollila 2021).

The latest public health policy paper formulated as government resolution (Valtioneuvosto 2021) and titled ‘Promotion of Wellbeing, Health, and Safety 2030’, linked security more tightly with the discussion on social integration and cohesion as well as health and health equity. More recently, the Ministry of Social Affairs and Health and broader social policy and public health community have sought to promote well-being economy as a key element of intersectoral policies, among others, by the development of the concept of and a proposal for indicators for well-being economy. However, this work has not been reflected in government policies or policy priorities in practice especially in relation to reduction of health and social inequalities.

The economic, commercial, and security policy priorities are high on the agenda of the current government. This is reflected in the enhanced role of the Ministry of Finance in oversight of social and health services, several decisions on increasing the availability of alcohol, and in relation to discussions on shifting the oversight on alcohol policies from the Ministry of Social Affairs and Health to the Ministry of Trade and Employment. While industry interference in policymaking is not new (Sama and Hiilamo 2019), it has been shown to have recently affected, e.g. the efforts of the current government to regulate nicotine pouches and to establish a health tax on sugary items (Koivusalo *et al*. 2025).

The aim of HiAP is to strengthen evidence-informed policymaking and accountability of policy decisions as regards the implications of policies across sectors for health, health equity, and health services. The lessons from Finland include that the wider regulatory and economic policy environment as well as other topical political priorities affect the opportunities and challenges for HiAP implementation. It is essential for the public health community to understand the changing political architecture and its priorities, adapt the implementation to the context, and find the existing windows of opportunities. Legal base offers some protection for maintaining the main structures and processes when political priorities change, but laws can always be altered.

The value of good public health capacities should not be underestimated. This should include public health expertise within the administration, strong public health institutions, and academia as well as consistent data production, analysis, and monitoring.

## Data Availability

No new data were generated for this editorial.
